# Supplementary material for: Chromothripsis during telomere crisis is independent of NHEJ, and consistent with a replicative origin
Source: Genome Res. 2019 May;29(5):737–49. doi: 10.1101/gr.240705.118 (PMC6499312; doi:10.1101/gr.240705.118)
Supplement: Supplemental Material [file supp_gr.240705.118_Supplemental_file_1.zip › contigs/annotated_contigs/DB84/contig.2.DB84_length_525_mean_cov_6.64761904762.docx]

**DB84_length_525_mean_cov_6.64761904762**

```
AATCAGATACAAGCACCATAAGCCTACTGGGGTTATGATAAAAGAGTGTCCTCACTGCACGTTGTAGGTATTGTAACCCAATTTTCTGA
 >chr16:78867196-78867479 - E=7e-148
ACTCGCAGAAGAGGACAATGATGAGGAAGGAGAATAGAAAGCAGGAGGAGGAGGAGGAGGAGGAGATGGCTGGGCCATTAAGTAAACAT

GAGAACAGCCACATTAGAGAAACAAGCTGTATGCAGGAAAGAGTGCAAGTTGAGGACTGGTATAAAGAAAGAGCTTGTCATCCCAGTGC

TTTGAGAGAA|GCACTCC|TTCTTGAGAAGGAGTTTCGCTCCTGCTGCCCAGGCTGGAGTGCAATGGTGCAATCTCGGCTCACCGCAAC
                  >chr16:79033131-79033372 - E=2e-127
TTCCGCCTTCTGGGTTGAAGCGATTCTCCTGCCTCAGCCTCTCGAGTAGCTGGGATTACAGGCATGTGCCACTACGCCTGGCTAATTTT

GTATTTTTAGTAGAGACAGGGTTTCACCATGTTGGTCAGGCTGGTCTCGAACTCCCGACCTCAGATGATCCGCCTGCATTGG
```
